# Cerebral Blood Flow during Rest Associates with General Intelligence and Creativity

**DOI:** 10.1371/journal.pone.0025532

**Published:** 2011-09-29

**Authors:** Hikaru Takeuchi, Yasuyuki Taki, Hiroshi Hashizume, Yuko Sassa, Tomomi Nagase, Rui Nouchi, Ryuta Kawashima

**Affiliations:** 1 Smart Ageing International Research Center, Institute of Development, Aging and Cancer, Tohoku University, Sendai, Japan; 2 Division of Developmental Cognitive Neuroscience, Institute of Development, Aging and Cancer, Tohoku University, Sendai, Japan; 3 Faculty of Medicine, Tohoku University, Sendai, Japan; 4 Department of Functional Brain Imaging, Institute of Development, Aging and Cancer, Tohoku University, Sendai, Japan; Freie Universitaet Berlin, Germany

## Abstract

Recently, much scientific attention has been focused on resting brain activity and its investigation through such methods as the analysis of functional connectivity during rest (the temporal correlation of brain activities in different regions). However, investigation of the magnitude of brain activity during rest has focused on the relative decrease of brain activity during a task, rather than on the absolute resting brain activity. It is thus necessary to investigate the association between cognitive factors and measures of absolute resting brain activity, such as cerebral blood flow (CBF), during rest (rest-CBF). In this study, we examined this association using multiple regression analyses. Rest-CBF was the dependent variable and the independent variables included two essential components of cognitive functions, psychometric general intelligence and creativity. CBF was measured using arterial spin labeling and there were three analyses for rest-CBF; namely mean gray matter rest-CBF, mean white matter rest-CBF, and regional rest-CBF. The results showed that mean gray and white matter rest-CBF were significantly and positively correlated with individual psychometric intelligence. Furthermore, mean white matter rest-CBF was significantly and positively correlated with creativity. After correcting the effect of mean gray matter rest-CBF the significant and positive correlation between regional rest-CBF in the perisylvian anatomical cluster that includes the left superior temporal gyrus and insula and individual psychometric intelligence was found. Also, regional rest-CBF in the precuneus was significantly and negatively correlated with individual creativity. Significance of these results of regional rest-CBF did not change when the effect of regional gray matter density was corrected. The findings showed mean and regional rest-CBF in healthy young subjects to be correlated with cognitive functions. The findings also suggest that, even in young cognitively intact subjects, resting brain activity (possibly underlain by default cognitive activity or metabolic demand from developed brain structures) is associated with cognitive functions.

## Introduction

Cerebral blood flow (CBF) during rest (rest-CBF) and cognitive functions among people with cognitive decline have been reported to be associated [1]. However, as the association between resting brain activity and cognitive functions among healthy young subjects has not been investigated, that investigation is the purpose of this study. In Hagberg and Ingvar [1]'s study, associations between cognitive reductions and decreases of the rest-CBF (especially the flow in the grey matter) was found. Those associations seemed to occur in regions having roles associated with the cognitive functions. Investigating the association between resting brain activity and cognitive functions has become increasingly important for the following reason. Resting brain activity has gathered a lot of scientific attention. Yet, research methods have focused on functional connectivity during rest (temporal correlations between BOLD signals in different brain regions during rest) or on the relative decrease of brain activity during the tasks, rather than on absolute resting brain activity. Regarding the magnitude of brain activity, the association between absolute brain activity during rest and cognitive functions certainly gives some insight into the findings from the analyses which used the relative decrease of brain activity in the task (described below).

Many recent neuroimaging studies have focused on a particular set of brain regions – the so called default mode network (DMN). These regions are deactivated during a wide range of externally directed, attention-demanding tasks (task induced deactivation; TID), rather than being deactivated during rest [2]. It has been stated that when an individual is awake and alert, yet not actively engaged in an attention-demanding task, a default state of brain activity exists [2]. Regions such as the lateral prefrontal cortex, part of the inferior parietal cortices, and the supplementary motor area are activated during cognitive tasks, whereas the regions forming the DMN [3] in the medial prefrontal cortices, the precuneus/posterior cingulate cortices, and parts of lateral parietal and lateral temporal cortices are deactivated [4].

TID in the DMN is associated with various cognitive factors and pathological states. The magnitude of TID in the DMN positively correlates with working memory capacity and decreases with aging [5]. Reduced TID in the DMN is observed in patients with autism, Alzheimer's disease and, schizophrenia [3], [6].

It is thought that investigating the association between cognitive factors and absolute resting brain activity, instead of TID, gives new insights into the association between default brain functions and cognition for the following reasons. First, regions that show relatively increased resting brain activity (measured by rest-CBF) and regions that show TID are partly similar, yet different [2]. Second, by measuring resting brain activity, it is possible to measure the resting brain activity of regions that do not belong to the DMN and do not show TID. Third, it is supposed that the magnitude of TID in the DMN reflects the cognitive load placed on subjects during the task [7] in addition to brain activities during rest. Furthermore, the magnitude of TID may also reflect the successful reallocation of cognitive resources [8]. Cognitive load and the reallocation of cognitive resources are important factors affecting brain activity, but they also warrant a distinct investigation of the association between cognitive factors and resting brain activity.

In order to examine the association between cognitive factors and measures of absolute resting brain activity, mean and voxel-by-voxel rest-CBF analyses were performed using arterial spin labeling (ASL; see Methods). The associations between rest-CBF and two essential components of cognitive functioning (namely psychometric general intelligence and creativity) were thus investigated. Creativity was measured by a divergent thinking test. Divergent thinking is said to be a key aspect of creativity [9] and it pertains primarily to information retrieval and the call for a number of varied responses to a certain item [10]. Creativity is associated with reduced TID in the DMN [11]; however, whether it relates to regional rest-CBF in the DMN is a matter of investigation. On the other hand, recent studies pointed out areas distributed across the brain (instead of certain specific regions) relating to general intelligence. Thus, we reasoned that mean rest-CBF may relate to general intelligence.

## Methods

### Ethics statement

In accordance with the Declaration of Helsinki (1991), Written informed consent was obtained from each subject. This study was approved by the Ethics Committee of Tohoku University.

### Subjects

Sixty-three healthy, right-handed individuals (32 men and 31 women) participated in this study as part of our ongoing project to investigate the association between brain imaging, cognitive functions, and aging [11], [12], [13], [14], [15], [16]. Data from these sixty-three subjects were used in our previous study to investigate the association between creativity and brain activity during a working memory task [11]. Note there are overlaps in data from the psychological data score in this study and our previous study [11]. All the subjects who took part in this study also became subjects of our intervention studies [17]. Psychological tests and MRI scans not described in this study were performed together with ones described in this study (Psychological data and imaging data gathered before the intervention were used in this study). The mean age of subjects was 21.6 years (standard deviation; SD, 1.68). All subjects were university students or postgraduate students. All subjects had normal vision and none had a history of neurological or psychiatric illness. Handedness was evaluated using the Edinburgh Handedness Inventory [18]. Data from one subject who failed to submit the questionnaire described below was excluded from the analysis.

### Assessment of general intelligence

Raven's advanced progressive matrix (RAPM) [19], a psychometric measure of general intelligence [19] which is often shown to be the measure most correlated with general intelligence [19], was used to assess general intelligence, as has been done in previous studies [12], [13]. General intelligence refers to the *g* factor [20], which contributes to success in diverse forms of cognitive tests regardless of whether they are verbal or nonverbal. The test contains 36 nonverbal items requiring fluid reasoning ability. The score of this test (number of correct answers in 30 min) was used as a psychometric index of individual intelligence. Alternatively or additionally, the scoring of this test may be possible based on the type of rule in the problem [21]. However, in this study, we focused on the total score of the test because it is highly unlikely that RAPM consists of more than a single factor, as summarized in Colom and Abad, [21]'s work. Also, use of the total score is by far the most widely used practice for this test.

### Creativity assessment

The S-A creativity test [22], which is a divergent thinking test, was used to assess creativity. A detailed discussion of the psychometric properties of this instrument and how it was developed is found in the test's technical manual [22]. The test was used to evaluate creativity through divergent thinking [22] and it involved three types of tasks. The first task required subjects to generate unique ways of using typical objects. The second task required subjects to imagine desirable functions in ordinary objects. The third task required subjects to imagine the consequences of ‘unimaginable things’ happening. The S-A creativity test provided a total creativity score, which was used in this study. It also scored the four dimensions of the creative process (fluency, originality, elaboration, and flexibility). In this study, the analysis was limited to total creativity score and did not include the score of each dimension because the score of each dimension highly correlated with the total creativity score. This is consistent with another group of rather similar DT tests [23]: Torrance Tests of Creative Thinking (TTCT) [24]. Heausler and Thompson [23] concluded that the correlations among the subscales in the TTCT were so high that each subscale could not provide meaningfully different information. Treffinger [25] warned that independent interpretations of the TTCT subscores should be avoided. Consistent with this notion, a previous study [26] that investigated the association between regional CBF and each dimension revealed that different creativity dimensions correlated with regional CBF in similar regions. Thus, we believe that using only the total creativity score serves the purpose of this study. Also, it is relatively uncommon for each dimension to be used in studies of the relationship between brain structures and psychometric measures of intelligence that focus on general intellectual ability rather than specific cognitive faculties [27]. For more details, including the psychometric properties of this test, validities of this test, sample answers to the questionnaire and the manner in which they were scored, see our previous works [12], [13].

### Mood assessment

In addition to cognitive functions, subject mood on the experiment day was also evaluated using the shortened Japanese version [28] of the profile of mood states (POMS) [29]. It contains 30 items from which Total Mood Disturbance can be calculated and used in the analyses. In our study, the POMS was used to measure each participant's mood on the experiment day. This measure was obtained to correct the effect of mood on rest-CBF, as mood has been shown to have an effect [30].

### Image acquisition

All MRI data acquisition was conducted with a 3-T Philips Intera Achieva scanner. ASL is an MR imaging method that allows noninvasive measurement of regional CBF. ASL was performed with quantitative signal-intensity targeting by alternating radio-frequency pulse labeling of arterial regions, which is a pulsed ASL method developed by Petersen et al.[31]. Details of the sequence and the calculation method for the perfusion parameters have been described elsewhere [31], [32], [33]. The actual imaging parameters were as follows: 64×64 matrix, TR = 300 ms, TE = 22 ms, FOV = 24 cm, 7 slices, 7.0 mm slice thickness (2.0 mm gap), SENSE = 2.5, 84 averages, scan duration 5 min 52 sec. We determined the position of the slice by putting the fourth of seven slices on the body of the corpus callosum in the coronal scout view [15]. During the scan of ASL, the subjects were instructed to keep still with their eyes closed, as motionless as possible, and not to sleep or think about anything in particular.

In addition to ASL, to obtain magnetization transfer ratio (MTR) maps, which represent a loss of signal due to the magnetization transfer effect, two successive scans, both with and without an additional RF magnetization transfer pulse, were acquired. The scan parameters were: TE = 3.1 ms, TR = 66 ms. FOV = 24 cm, 1.25×1.25×2 mm^3^ voxels, 70 slices, 2.0 mm slice thickness. Scan duration was 6 min 39 sec. Finally, using a magnetization-prepared rapid gradient-echo Imaging sequence, high-resolution T1-weighted structural images (240×240 matrix, TR = 6.5 ms, TE = 3 ms, FOV = 24 cm, 162 slices, 1.0 mm slice thickness) were collected. These MTR maps and T1-weighted structural images were used for the normalization of the regional CBF maps. These scans, as well as other scans used in another study [11], were in most cases taken during the same session.

### Preprocessing of imaging data

Maps of rest-CBF and the longitudinal relaxivity (R1) of each subject were obtained by using dedicated software running on IDL (Research Systems, Boulder, Colo), which was developed and provided by Petersen et al. [31] (National Neuroscience Institute, Singapore). The following constants were used in the CBF calculation: T1 of arterial blood, 1.65 seconds; inversion efficiency, 95%; blood-brain partition coefficients for gray and white matter, 0.98 and 0.82, respectively. For an explanation of the details and technicalities of regional CBF calculation, see Petersen et al. [31] and Yoshiura et al. [34]. The mean CBF of the gray matter and the mean CBF of the white matter of each subject were obtained with this software. The resulting mean CBF of the gray matter and mean CBF of the white matter were then forwarded to the group regression analysis described below.

Preprocessing and data analysis were performed using Statistical Parametric Mapping software (SPM5; Wellcome Department of Cognitive Neurology, London, UK) implemented in Matlab (Mathworks Inc., Natick, MA, USA). Prior to the normalization of the regional CBF map, an original template of the T1-weighted structural image was created as follows. (a) The T1-weighted structural image of each subject was spatially normalized to the T1 template in SPM5. (b) The normalized T1-weighted structural image of each subject was then smoothed using a Gaussian kernel of 8 mm FWHM and finally averaged across subjects to make an original template of the T1-weighted structural image. Previous voxel-based morphometry (VBM) and fMRI studies have shown the usefulness of making a study-specific template for the normalizing procedure from the normal subject group [35], [36].

Prior to MTR calculation, the magnetization transfer image volume acquired with the saturation pulse (Sat) was linearly registered to the magnetization transfer image volume acquired without the saturation pulse (NoSat). The MTR image volume was calculated by: 100 * (NoSat−Sat)/NoSat. Then, the R1 map of each subject, which maintained its alignment with the regional CBF map of each subject, was coregistered to the MTR image with the within-subject registration method (the coregisteration option in SPM) [37] using the mutual information option. This within –subject registration method was used throughout the processes of coregistration in this study. The MTR image of each subject, which maintained its alignment with both the regional CBF map and R1 map of each subject, was next coregistered to the T1-weighted structural image of each subject. The T1-weighted structural image of each subject was normalized to our original template of the T1-weighted structural image. Using the parameters for this normalizing procedure, the MTR image and regional CBF map of each participant was spatially normalized to create images with 1.25-* 1.25- * 1.25-mm^3^ voxels. The MTR image was then segmented using SPM5's segmentation option. Next, a gray matter mask image that consisted of voxels whose values were higher than 0.2 in the gray matter density image, resulting from the segmentation of the MTR image, was generated. This gray matter mask image was applied to the regional CBF map to limit the analysis so as to generate the gray matter regional CBF map. Also, this gray matter mask image was applied to the segmented gray matter (density) image to generate the regional gray matter density (rGMD) map, which has the same gray matter area as the gray matter regional CBF map generated above. These processed normalized gray matter regional CBF maps and (masked) rGMD maps were then spatially smoothed using a Gaussian kernel of 8 mm FWHM. The resulting maps, representing the gray matter regional CBF and (masked) rGMD maps (which have the same gray matter area as the gray matter regional CBF maps), were forwarded to the group regression analysis described below.

Both the MTR image and the T1-weighted structural image were used for preprocessing for the following reasons, (a) The T1-weighted structural image has the most refined solution and is suited for normalizing procedures. However, the contrast of our T1 weighted structural image is not close to the R1 image. It is probably for this reason that the within-subject registration between the T1 weighted structural image and the R1 image did not work in several cases, as was confirmed by visual inspection. Furthermore, our T1 weighted structural image is not compatible with SPM5/VBM5's segmentation. The segmentation of this image with SPM5/VBM5 sometimes results in apparent segmentation errors, which are visible on first sight from visual inspection. (b)The R1 image's resolution was not good and the R1 image taken with our ASL sequence does not show the whole brain structure. It is best suited for neither normalization nor segmentation. We confirmed that these processes (segmentation and normalization) using R1 images often failed through visual inspection. (c) The MTR image is close to both the R1 image and the T1-weighted structural image and allows itself to be coregistered with both the R1 image and the T1 weighted structural image. Additionally, it is compatible with SPM5's segmentation. We confirmed these processes did not fail (at least the failures were not apparent at first sight) in all subjects, through visual inspections.

### Statistical analysis of behavioral data and mean rest-CBF

The behavioral data and data of the mean rest-CBF of the gray and white matter were analyzed using the statistic software SPSS 18.0 (SPSS Inc., Chicago, IL). Associations among psychological data (RAPM score, total score of the S-A creativity test, POMS score, sex, and age) were analyzed using simple regression analyses. We used a multiple linear regression analysis to investigate whether each of the mean rest-CBFs of the gray and white matter was significantly related to RAPM score, total creativity score in the S-A creativity test, total mood disturbance score of the POMS, age, and sex. In these analyses, dependent variables were the mean rest-CBF value of gray and white matter, respectively. The independent variables were RAPM score, total creativity score in the S-A creativity test, total mood disturbance score of the POMS, age, and sex.

### Statistical Analysis of regional rest-CBF

At the group level analysis, we tested for a relationship between cognitive functions (general intelligence and creativity) and regional rest-CBF. In the whole brain analysis, we used a multiple linear regression analysis to look for areas where the regional rest-CBF was significantly related to individual general intelligence (RAPM score) and individual creativity as measured by the divergent thinking test (total creativity score in the S-A creativity test). The analysis was performed with sex, age, and the score of POMS as additional covariates. By using SPM5's ANCOVA option of global normalization and setting the grand mean scale value at 50 ml/dl/min, we corrected the confounding effect of differences in mean (gray matter) CBF.

### Statistical Analysis of regional rest-CBF, correcting the effect of regional gray matter structure

In the abovementioned whole brain regional rest-CBF analysis, the effect of the regional gray matter structure on regional-rest CBF was not corrected. This was done so that our studies could be associated with relevant previous studies. Gray matter distributions were also not taken into account in PET studies, nor in the other functional MRI studies [2], [11] we referred to. However, to rule out the possibility that regional gray matter structure affected the significance of the findings, we also performed another analysis that takes into account the effect of rGMD. To that end, we used the biological parametric mapping (BPM) toolbox for SPM [38]. The BPM toolbox can handle voxel-by-voxel correlations between multi-modal images. It has been used to correct the effect of regional gray matter structures on functional activation [39]. In this multiple regression analysis, the dependent variable at each voxel was the value of the (gray matter) regional rest-CBF at the voxel. The independent variables at the voxel were the signal intensity (at the voxel) of the rGMD image created above, age, sex, the score of POMS, mean gray matter rest-CBF, general intelligence (RAPM score), and individual creativity, as measured by the divergent thinking test (total creativity score in the S-A creativity test).

### Statistical threshold

Regions with significance were inferred using cluster-level statistics [40]. In this procedure, the null hypothesis was rejected when the clusters had a large spatial extent. The distribution of cluster sizes can be found by parametric methods based on the theory of Gaussian random fields, which accounts for image volume, smoothness, and the cluster defining threshold. At the cluster level, inference is done at the cluster size; that is the probability that any cluster larger than the critical cluster size is controlled. Only clusters with a *P*<0.05, after correction for multiple comparisons at cluster size with a voxel-level cluster-determining threshold of *P*<0.005 uncorrected, were considered statistically significant in this analysis.

## Results

### Behavioral data

**Table 1** shows the Average and SD of scores from the S-A creativity test, RAPM score, Profile of Mood States (POMS; see Methods) score (Total Mood Disturbance) and age. Neither the RAPM score, the total S-A creativity test score, age, sex, nor the POMS score correlated with each other (*P*>0.05, simple regression analysis).

**Table 1:** Average and SD of scores from the S-A creativity test, RAPM score, POMS score (Total Mood Disturbance) and age in our sample.

	average	SD	range
S-A creativity test score	38.8	11.1	7–60
RAPM score	27.3	3.91	18–35
POMS score (Total Mood Disturbance)	7.2	11.8	−7–51
age	21.5	1.66	18–27

The POMS score was not obtained from one subject among the 63 subjects who participated in this study. It was removed from the analysis and the data in this table is data from the remaining 62 subjects.

### Correlation of mean gray and white matter rest-CBF and cognitive functions

**Figure 1:**
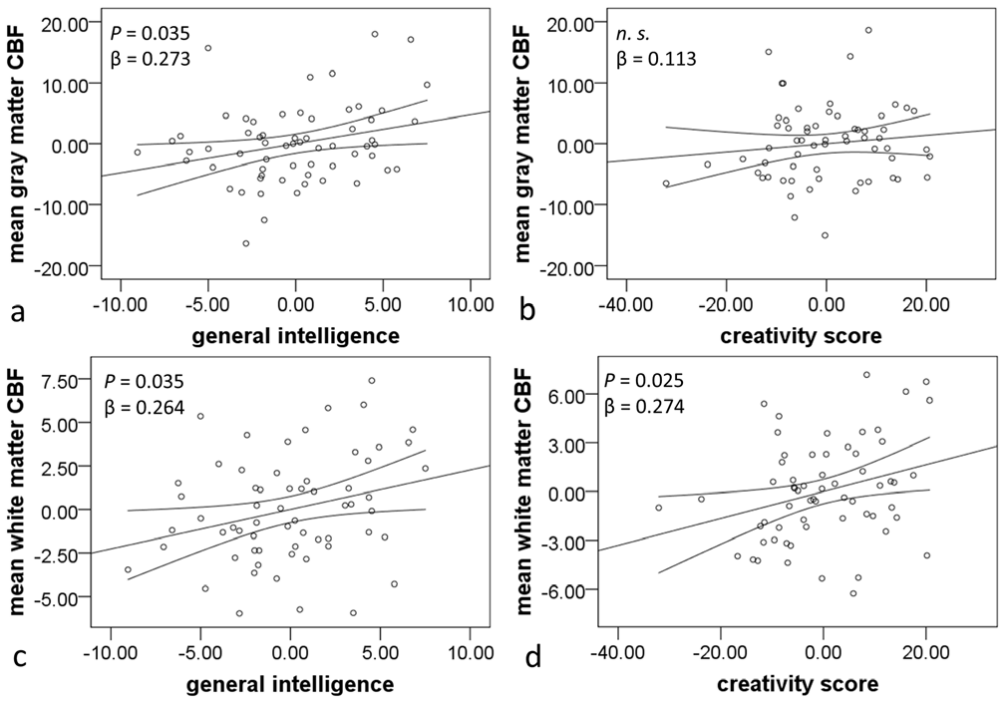
Associations between mean gray and white matter rest-CBF and cognitive functions. Residual plots with trendlines depicting the correlations between residuals in the multiple regression analyses with mean gray/white matter rest-CBF as the dependent variable and five other variables (RAPM score, the total S-A creativity test score, age, sex, and the POMS score) as independent variables. 95% confidence intervals for the trendlines were shown. (a) The association between mean gray matter rest-CBF and RAPM score. (b) The association between mean gray matter rest-CBF and the total S-A creativity score. (c) The association between mean white matter rest-CBF and the RAPM score. (d) The association between mean white matter rest-CBF and the total S-A creativity score.

A multiple regression analysis with mean gray mater rest-CBF as the dependent variable and five other variables (RAPM score, the total S-A creativity test score, age, sex, and the POMS score) as independent variables was performed. The analysis revealed that the RAPM score was significantly and positively correlated with mean gray matter rest-CBF (Fig. 1a). However, in this analysis, the total S-A creativity test score was not correlated with mean gray matter rest-CBF (Fig. 1b). The analysis also revealed that the POMS score was significantly and positively correlated with mean gray matter rest-CBF (disturbed mood was associated with higher mean rest-CBF). However, sex (male = 0, female = 1 in this analysis) was not significantly associated with mean gray matter rest-CBF. Statistical values for this analysis are shown in **Table 2**.

**Table 2:** Statistical values of the multiple regression analysis with mean gray matter rest-CBF as the dependent variable and five other variables [RAPM score, the total S-A creativity test score, age, sex (male = 0, female = 1), and the POMS score] as independent variables.

	P value	t value	Non standardized partial regression coefficient (B)	95% confidence prediction intervals of B	Standardized partial regression coefficient (β)
RAPM score	0.035	2.16	0.476	0.035≤B≤0.917	0.273
S-A creativity test score	0.364	0.92	0.069	−0.082≤B≤0.220	0.113
POMS score	0.037	2.13	0.156	0.009≤B≤0.302	0.271
Age	−0.034	−0.07	0.949	−1.086≤B≤1.018	−0.008
Sex	0.053	1.975	3.371	−0.048≤B≤6.791	0.249

Then, a multiple regression analysis with mean white mater rest-CBF as the dependent variable and five other variables (RAPM score, the total S-A creativity test score, age, sex, and POMS score) as independent variables was performed. The analysis revealed that the RAPM score was significantly and positively correlated with mean white matter rest-CBF (Fig. 1c). Also, the total S-A creativity test score was significantly and positively correlated with mean white matter rest-CBF (Fig. 1d). The analysis also revealed that the POMS score was not significantly correlated with mean white matter rest-CBF. However, sex (male = 0, female = 1 in this analysis) was not significantly associated with white matter rest-CBF. Statistical values for this analysis are shown in **Table 3**.

**Table 3:** Statistical values of the multiple regression analysis with mean white mater rest-CBF as the dependent variable and five other variables [RAPM score, the total S-A creativity test score, age, sex (male = 0, female = 1), and the POMS score] as independent variables.

	P value	t value	Non standardized partial regression coefficient (B)	95% confidence prediction intervals of B	Standardized partial regression coefficient (β)
RAPM score	0.035	2.16	0.227	0.016≤B≤0.437	0.264
S-A creativity test score	0.025	2.30	0.083	0.011≤B≤0.155	0.274
POMS score	0.055	1.96	0.068	−0.001≤B≤0.138	0.242
Age	0.750	−0.32	−0.080	−0.582≤B≤0.422	−0.040
Sex	0.078	1.79	1.461	−0.170≤B≤3.092	0.220

The association between negative mood and increased rest-CBF in healthy subjects is consistent with previous studies [30].

### Correlation of regional rest-CBF and general intelligence (RAPM score)

**Figure 2:**
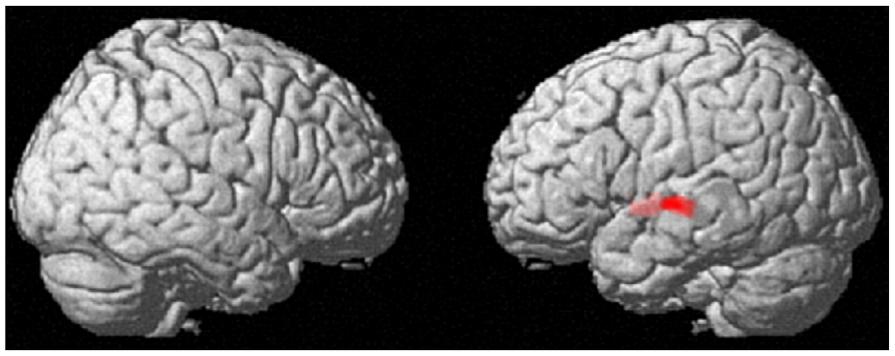
The region of significant positive correlation between regional rest-CBF and general intelligence (RAPM score). The region with significant correlation is seen in the anatomical cluster that includes the left superior temporal gyrus, insula and contingent regions (*P* = 0.036 corrected for multiple comparisons at cluster size, with a voxel-level cluster-determining threshold of *P*<0.005 uncorrected).

A whole brain multiple regression analysis was then performed. After controlling for the total S-A creativity test score, the POMS score, age, sex, and mean gray matter rest-CBF, it became evident that the RAPM score was significantly and positively correlated with regional rest-CBF in a perisylvian cluster. This perisylvian cluster included the superior temporal gyrus, insula, and contingent regions (*x*, *y*, *z* = −53, −16, −1; *t* = 3.62; *P* = 0.036, corrected for multiple comparisons at cluster size with a voxel-level cluster-determining threshold of *P*<0.005, uncorrected. Fig. 2). No regions showed significant negative correlations.

The significance of this cluster did not change when the effect of rGMD was corrected at every voxel (*x*, *y*, *z* = −34, −4, −4; *t* = 3.51; *P* = 0.006, corrected for multiple comparisons at cluster size). In this analysis in which the effect of rGMD was corrected at every voxel, there were no other significant results.

### Correlation of regional rest-CBF and creativity (total S-A creativity test score)

**Figure 3:**
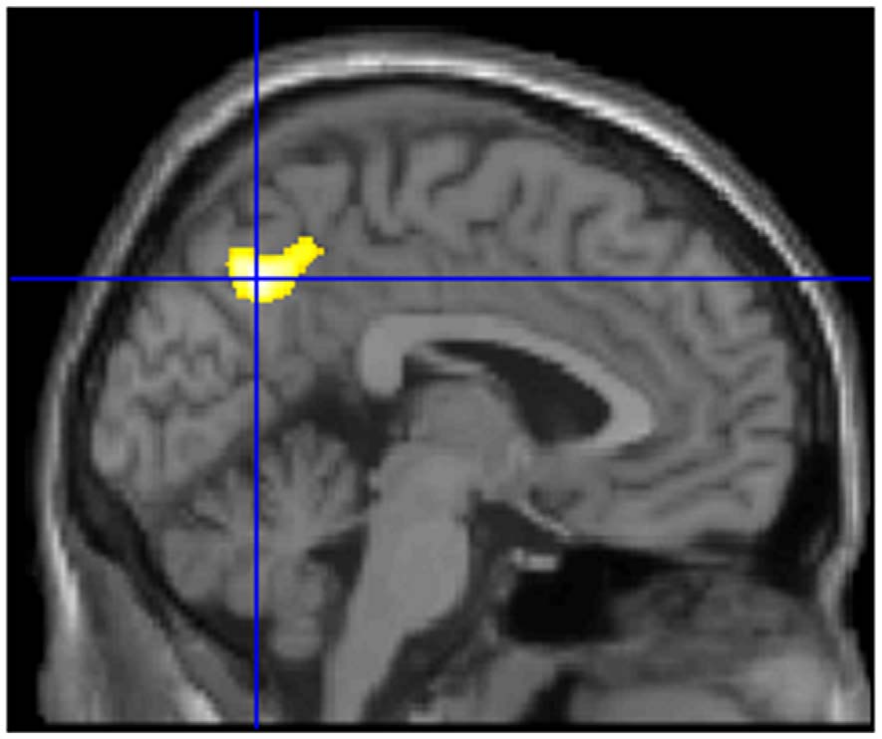
The regions of significant positive correlation between regional rest-CBF and creativity (the total score of S-A creativity test). The region with significant correlation is seen in the precuneus (*P* = 0.050 corrected for multiple comparisons at cluster size, with a voxel-level cluster-determining threshold of *P*<0.005 uncorrected).

After controlling for the RAPM score, the POMS score, age, sex, and the mean gray matter rest-CBF, the analysis also revealed that the total S-A creativity test score was significantly and negatively correlated with regional rest-CBF in the precuneus (*x*, *y*, *z* = −3, −64, 40; *t* = 3.98; *P* = 0.050, corrected for multiple comparisons at cluster size. Fig. 3). No regions showed significant positive correlations.

The significance of this cluster did not change when the effect of rGMD was corrected at every voxel (*x*, *y*, *z* = −1, −65, 41; *t* = 3.74; *P* = 0.007, corrected for multiple comparisons at cluster size). In this analysis, there were no other significant results.

## Discussion

To the best of our knowledge, this is the first study to investigate the association between rest-CBF and cognitive functions in healthy young subjects. The findings showed mean and regionally specific rest-CBF in healthy young subjects to be correlated with cognitive functions. The findings suggest, even in young cognitively intact subjects, resting brain activity (possibly underlain by default cognitive activity or metabolic demand from developed brain structures) is associated with cognitive functions. Consistent with our hypothesis, our findings showed that mean gray and white matter rest-CBF were significantly and positively correlated with individual psychometric intelligence as measured by RAPM. Furthermore, after correcting the effect of mean rest-CBF, regional rest-CBF in the precuneus showed a significant negative correlation with individual creativity. This negative correlation between creativity and regional rest-CBF in the precuneus may at least partly underlie the association between creativity and reduced TID in the DMN [11].

In this study, increased mean gray and white matter rest-CBF were associated with higher general intelligence. This finding is congruent with the notion that areas distributed across the brain are associated with general intelligence. Increased rest-CBF may be necessary for maintaining cortical structures rich in cell components in subjects with higher general intelligence. Recent studies [27], [41] have pointed out brain structures in areas distributed throughout the brain (instead of in certain specific regions such as the prefrontal cortex) relating to general intelligence. Current findings of the association between mean rest-CBF and general intelligence are congruent with this notion. These findings also present a steep contrast to the neural efficiency theory which posits that increased general intelligence is associated with efficient cognitive processes and reduced neural activity [42]. Absolute metabolic rate during the cognitive tasks is negatively correlated with psychometric intelligence [43]. Adding these previous findings and our results together shows that higher psychometric intelligence is associated with generally reduced brain activity during cognitive tasks and increased brain activity during rest. Such a notion suggests that neural efficiency in highly intelligent subjects is limited to neural activity during task execution and is not extended to intrinsic brain activity itself.

There was a significant positive correlation between general intelligence and increased regional rest-CBF in the left-side perisylvian region. This results is congruent with some previous studies on the neural basis of psychometric intelligence and may indicate high facilitation of language processes in highly intelligent subjects. Increased regional rest-CBF in the left perisylvian region, (an anatomical cluster that includes the left superior temporal gyrus, insula and contingent regions) is associated with higher general intelligence after the effect of mean rest-CBF has been removed. This finding is congruent with the report that higher psychometric intelligence is associated with functional activity in the left-side perisylvian region during a language-related task [44] and cortical thickness in the left-middle to superior temporal gyrus [45]. The left-side perisylvian region is consistently activated in response to language processes and is thus considered to play a key role in language processes [46]. Perhaps, highly intelligent subjects recruite more language processes during default cognitive processes.

Increased mean white matter rest-CBF (but not mean gray matter rest-CBF) in creative subjects may be necessary for maintaining the myelinated white matter structures of creative subjects. As for creativity, a positive correlation between mean white matter rest-CBF (and not mean gray matter rest-CBF) was observed. The correlation between cognitive functions and rest-CBF in white matter observed in this study is congruent with that of people with cognitive decline [1] and people with cerebral microangiopathy [47] reported in previous studies. Traditionally it has been suggested that brain connectivity plays a key role in creativity [48], [49]. Our previous diffusion tensor imaging study [12] reported that increased wide-spread white matter structural integrity, which is presumably secondary to increased myelination [50], is associated with higher creativity. Increased origodndroglia, which are responsible for the metabolic maintenance of the increased myelin sheath [51], may require increased rest-CBF to keep their functions. In that way, creativity may be associated with increased mean white matter rest-CBF. Conversely, in vascular dementia of Binswanger's type, in which reduced blood supply in white matter is seen, loss of oligodendrocytes, nerve fiber loss, and thin myelin sheaths in the white matter are observed [52], [53]

Decreased regional rest-CBF in the precuneus in creative subjects is congruent with (a) clinical studies that reported an association between resting brain activity and pathology and (b) studies that reported an association between creativity and pathology. Additionally, it may have roots in creative subjects' poor ability in social functioning. This pattern of rest-CBF in creative subjects is distinct from the pattern of rest-CBF in the highly intelligent subjects observed in this study, supporting the distinction between creativity and psychometric intelligence. Previous studies have reported reduced TID in the DMN in patients with schizophrenia and their relatives, and also in patients with autism spectrum disorders [3], [6]. Previous studies also reported associations between the reduced resting glucose metabolism of the DMN in patients with autism spectrum disorders [3]. Furthermore, creativity has been consistently shown to be associated with psychopathologies including schizotypy [54]. The association between creativity measured by divergent thinking tests and patients with autistic spectrum disorders is not known, however such patients are known to have a high systemizing quotient [55]. Systemizing is the drive to analyze, explore and construct a system to provide a way of understanding and predicting the behavior of non-agents [55]. It entails extracting the underlying rules that govern a system, and not only allows us to understand and predict an existing system, but to invent a new one. In addition the high ability to systemize, creativity is also associated with impaired social skills [22]. On the other hand, the DMN is involved in internally focused tasks and social cognition including autobiographical memory retrieval, envisioning the future, and conceiving the perspectives of others [3]. The precuneus is an important node in the DMN as it is where subsystems of the DMN are thought to converge [3]. Thus, reduced regional rest-CBF in the precuneus in creative subjects, which is comparable to aberrant resting activity in the DMN in patients with certain diseases, may have roots in creative subjects' impaired social skills. Without relying on anecdotal evidence, studies have consistently identified creativity (irrespective of whether or not it is measured by divergent thinking tests) as being associated with impaired social skills, less sociability, and certain psychopathologies [22], [54], [56]. Perhaps, reduced regional rest-CBF in the precuneus in creative subjects is one of the neural mechanisms that underlies this phenomenon. It may be compensating the cognitive resources of regions outside the DMN.

Alternatively, reduced regional rest-CBF in the precuneus may have the same mechanism which was suggested to explain the reduced TID in this region during a working memory task [11]. We suggested inefficient reallocation of cognitive resources (inattention) might underlie the reduced TID in the precuneus during a working memory task in creative subjects in our previous study [11]. A similar mechanism may underlie reduced regional rest-CBF. Attention is characterized by an increase of brain activity in the regions relevant to the cognitive processes attended to and a decrease of brain activity in the regions irrelevant to said cognitive processes [57]. Thus, reduced rest-CBF in the precuneus in creative subjects may be a reflection of their inattention. Creative subjects may not be able to concentrate cognitive resources on default cognitive processes which are ongoing during rest and in which the precuneus played a central role [3]. Those cognitive resources may be used in miscellaneous cognitive processes (such as distraction by external stimuli) and that may be reflected in reduced resting regional rest-CBF in the precuneus. However, this idea is speculative and needs to be confirmed by future experiments.

Our previous study reported that reduced TID in the precuneus during a working memory task is associated with higher creativity [11]. In that study, we discussed the findings mainly in terms of impaired selective attention (or deactivation failure in regions irrelevant to task execution) and did not attribute the reduced TID in the precuneus to reduced resting brain activity in the precuneus. However, our current findings indicate that reduced TID in the precuneus may at least be partly attributed to reduced resting brain activity in the precuneus.

There are a few limitations in this study. We used young healthy subjects with a high-level of education (university students). As was the case with our previous studies [12], [13], limited sampling of the full range of intellectual abilities is a common hazard when sampling from college cohorts. Whether our findings would also hold across the full range of population samples and normal distribution must be determined with larger and more representative samples. However, given the correlation between intelligence and creativity among subjects with normal and inferior intelligence [58], focusing on highly intelligent subjects was certainly warranted for the purpose of this study. Furthermore, our ASL sequence has low spatial resolution, and it is sensitive to subjects' motion [32]; these factors might lead to reduced sensitivity in the analysis of regional rest-CBF. Finally, in this study, the present results were only statistically marginally significant, although association between regional rest-CBF and creativity was seen in the region having an *a priori* hypothesis. Our statistical procedures are standard and widely used in analyses of mean rest-CBF and of regional rest-CBF (multiple regression analyses and, in the case of the regional rest-CBF analysis, cluster-level statistics [40] for corrections of multiple comparisons were employed). However, there were many analyses in this study, and across the studies of this project, which can falsely increase risk of rejecting the null hypothesis with an increased number of tests. Thus, the results should be taken cautiously until replicated.

This is the first study to investigate the association between rest-CBF and cognitive functions among healthy young subjects. Recent neuroimaging studies focused on the associations between resting brain activity and cognition using the paradigm of TID and functional connectivity between different brain regions during rest. Our findings showed that mean and regionally specific rest-CBF during rest in healthy young subjects correlated with cognitive functions. The significance of the regional rest-CBF results did not change when the effect of rGMD was corrected. The findings suggest that, even in young cognitively intact subjects, resting brain activity (possibly underlain by default cognitive activity or metabolic demand from developed brain structures) is associated with cognitive functions.
